# Biophysical approaches for the study of interactions between molecular chaperones and protein aggregates

**DOI:** 10.1039/c5cc03689e

**Published:** 2015-08-10

**Authors:** Maya A. Wright, Francesco A. Aprile, Paolo Arosio, Michele Vendruscolo, Christopher M. Dobson, Tuomas P. J. Knowles

**Affiliations:** Department of Chemistry, University of Cambridge Lensfield Road Cambridge CB2 1EW UK tpjk2@cam.ac.uk +44 (0)1223 336300

## Abstract

Molecular chaperones are key components of the arsenal of cellular defence mechanisms active against protein aggregation. In addition to their established role in assisting protein folding, increasing evidence indicates that molecular chaperones are able to protect against a range of potentially damaging aspects of protein behaviour, including misfolding and aggregation events that can result in the generation of aberrant protein assemblies whose formation is implicated in the onset and progression of neurodegenerative disorders such as Alzheimer's and Parkinson's diseases. The interactions between molecular chaperones and different amyloidogenic protein species are difficult to study owing to the inherent heterogeneity of the aggregation process as well as the dynamic nature of molecular chaperones under physiological conditions. As a consequence, understanding the detailed microscopic mechanisms underlying the nature and means of inhibition of aggregate formation remains challenging yet is a key objective for protein biophysics. In this review, we discuss recent results from biophysical studies on the interactions between molecular chaperones and protein aggregates. In particular, we focus on the insights gained from current experimental techniques into the dynamics of the oligomerisation process of molecular chaperones, and highlight the opportunities that future biophysical approaches have in advancing our understanding of the great variety of biological functions of this important class of proteins.

## Abstract 2

We give an overview of current biophysical methods that are being used to study molecular chaperones and their interactions with amyloid proteins.

## Graphical abstract

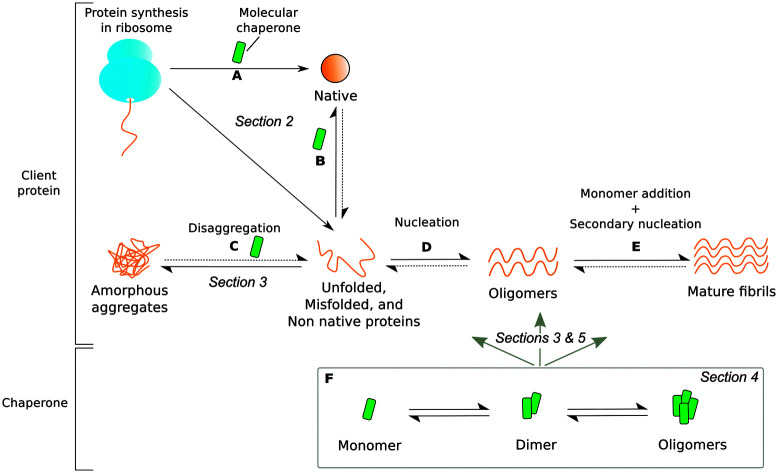

## 1 Protein folding and aggregation

Proteins take part in virtually all of the key molecular processes in living organisms by interacting with a wide range of biomolecules, including other proteins. In order to function properly, proteins generally have to fold into specific three-dimensional structures known as native states. The folding of proteins into these conformations is, at least at low concentrations, a spontaneous self-assembly process in which their free energy is minimised,^1^ through the optimization of intramolecular interactions within the protein chain as well as protein–solvent interactions.^1–3^ In particular, it is generally favourable for amino acid residues with hydrophobic side chains to be buried within the folded native structure, shielded from the aqueous cell medium.^3^ However, at higher concentrations, such as those found in the cellular environment, the native state can become metastable relative to self-assembly into aggregates.^4–6^ In addition, incompletely formed nascent protein chains emerging from the ribosome are typically exposed to the highly crowded cellular environment in an unfolded state for long periods with respect to the time required for their folding.^7^ Indeed, slow rates of codon translation significantly increase the chance of misfolding through inappropriate intramolecular interactions between hydrophobic amino acid residues on nascent chains, and between nascent chains and other proteins or biomolecules within the cytoplasm.^7,8^ In order to counteract protein misfolding, cells contain a range of protective mechanisms, notably molecular chaperones, which assist proteins in attaining their native conformations. However, some proteins escape these control mechanisms and form partially folded or misfolded states which lack their intended biological function and which are particularly prone to aggregation.^7^

Protein aggregates can range from amorphous species to highly ordered β-sheet rich amyloid fibrils. Amorphous aggregates are largely disordered deposits and can be degraded readily within the cell,^9,10^ whereas amyloid aggregates are highly persistent and stable assemblies of proteins. The formation of such aggregates is associated with the onset and development of a range of disorders such as Alzheimer's and Parkinson's diseases, type II diabetes, and the formation of eye lens cataracts.^11–14^ The mechanism by which soluble monomeric proteins assemble into fibrils involves several distinct microscopic processes.^15^ Typically, protein monomers first assemble into soluble low molecular weight oligomers through primary nucleation. These oligomers then elongate *via* monomer addition, leading to the formation of long and highly organised mature fibrils. Amyloid formation is accelerated by fragmentation of existing fibrils and other forms of secondary events, for example secondary nucleation in which the surfaces of mature fibrils catalyse the nucleation of monomers as a feedback system that results in the production of new aggregates.^15,16^ To counteract the numerous pathways that lead to the formation of fibrils, it is becoming increasingly clear that cells utilise molecular chaperones as a front line defense mechanism against amyloid formation.

## 2 Molecular chaperones assist in protein folding

The cell and its extracellular environment possess a complex system of molecular chaperones that intervene in various steps along the protein folding pathways. The first molecular chaperones encountered by proteins are those associated with the ribosome.^7^ In eukaryotes, such molecular chaperones include the nascent chain-associated complex (NAC) and the ribosome-associated complex (RAC), which are bound directly to the ribosome and dynamically interact with nascent chains during protein biogenesis.^7,17–19^ NAC exists as a heterodimer of α and β components, and it is thought to shield hydrophobic residues on protein chains emerging from the ribosome.^7,18,20^ RAC is a multiprotein complex that stabilises nascent chains and, with the help of other downstream molecular chaperones, assists around 20% of proteins in attaining their native conformations.^17^ Protein chains that cannot reach their native states with the help of NAC and RAC continue to elongate, and encounter the nonribosome-binding Hsp70/Hsp40 system which acts further downstream in the protein folding process.^21^ In an ATP-dependent cycle, Hsp70 rapidly binds and unbinds to hydrophobic patches on nascent protein chains to prevent inappropriate interactions, and to decrease the concentration of aggregation prone unfolded species.^7^ During this process, Hsp40, a 40 kDa heat shock protein, acts as a cochaperone that increases the efficiency of the ATP binding and release cycle.^22^ Finally, upon release from the ribosome, protein chains that still need assistance in folding proceed to interact with the TriC (TCP1 ring complex) or Hsp90 chaperone system.^17,23^ TriC operates by encapsulating non-native protein chains in a chamber and providing a shielded environment in which they can fold undisturbed.^23^

## 3 Molecular chaperones curtail amyloid formation

**Fig. 1:**
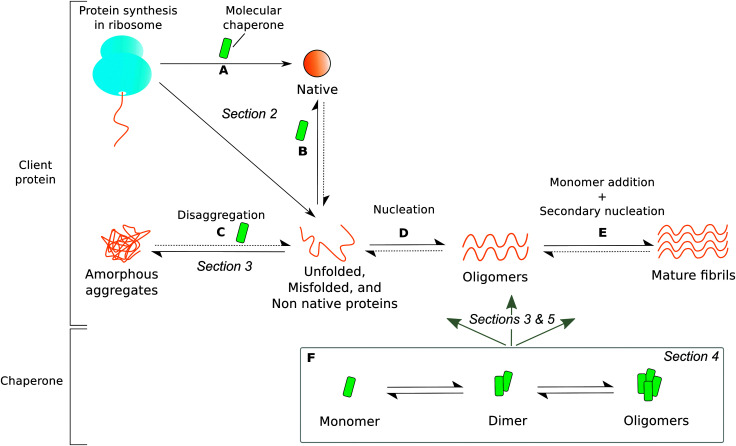
The roles of molecular chaperones (green) in protecting proteins from aggregation. Dotted arrows signify very slow processes. The section in which each type of molecular chaperone function is discussed is labelled in italics. Molecular chaperones are able to (A) protect ribosome-bound nascent chains and assist in co-translational folding, (B) rescue and refold unfolded, misfolded, or non native proteins released from the ribosome, (C) disaggregate amorphous deposits, (D) interact with pre-fibrillar amyloidogenic proteins, and (E) bind to mature fibrils. However, the dynamic oligomeric states of molecular chaperones (F) contribute greatly to the heterogeneity of chaperone-substrate systems by interacting with species present in all processes (A) through (E). Partially adapted from ref. 24.

The role that molecular chaperones play in assisting protein folding has been studied extensively, and significant progress has been made in elucidating the mechanisms involved, as described in the previous section. However, increasing evidence indicates that assisting nascent protein chains in reaching their native conformations is just one of the many functions of molecular chaperones (Fig. 1A). Indeed, several studies have shown that molecular chaperones also play a more direct role in protecting against protein aggregation by refolding non native proteins and promoting disaggregation of misassembled proteins (Fig. 1B and C), as well as inhibiting fibril formation by establishing interactions with a variety of amyloidogenic species such as partially folded intermediates, toxic oligomers, and mature amyloid fibrils (Fig. 1D and E).^24–34^ For example, the extracellular molecular chaperones α_2_-macroglobulin and clusterin have been shown to interact with a variety of amyloidogenic proteins to inhibit the formation of fibrils.^35–37^ At the same time, there exist many different molecular chaperones that act predominantly inside the cell to prevent a range of aberrant processes, including those associated with the progression of neurodegenerative diseases.^38^ In the following we discuss specifically the function of two ubiquitous types of intracellular eukaryotic molecular chaperones, Hsp70 and αB-crystallin (αBC), which have been popular targets of biophysical characterisation studies because of their protective role against protein aggregation and their oligomerisation properties.

Human Hsp70 has been shown to inhibit, at substoichiometric ratios, the formation of α-synuclein and amyloid β(1–42) peptide aggregates (Aβ_42_) which are associated with Parkinson's and Alzheimer's diseases, respectively.^33,39–41^ Such inhibitory action has not only been observed in human cells, but also in yeast where Hsp70 family members have been shown to increase the lag time in the aggregation of Ure2p, a prion protein.^42^ Although it has been proposed that Hsp70 acts by binding to aggregation-prone monomeric proteins to suppress nucleation in human cells,^33^ this has not been experimentally confirmed and the mechanism of chaperone action still remains largely unknown.

Members of the Hsp70 family are also known for their ability to reverse the formation of protein aggregates with the assistance of co-chaperones both *in vivo* and *in vitro*.^24–30^ For example, the Hsp104, Hsp70, and Hsp40 systems have been shown to disaggregate and refold firefly luciferase, β-galactosidase clusters,^25^ and heat denatured green fluorescent protein (GFP).^29^ The proposed mechanism of disaggregation involves first the identification of protein aggregates by Hsp70 and Hsp40 in an ATP-dependent process. Then, Hsp70 and Hsp40 bind and extract individual misfolded protein molecules from the surface of these clusters.^24,29,43^ Subsequently, the extracted misfolded molecule is translocated across ClpB (an Hsp104 homologue), which allows it to unfold and exit into the cytoplasm, where it achieves its native conformation spontaneously or with the help of other molecular chaperones.^24,27,43^ Disaggregation by Hsp104 has been observed for both amorphous and amyloid aggregates.^24,44^ The variety of molecular chaperone functions that Hsp70 possesses, including preventing protein misfolding, inhibiting amyloid fibril growth, and resolubilizing aggregates, makes it a key part of the network of molecular defences in place for assisting cells in combating protein aggregation.

Another crucial molecular chaperone known for its ubiquity and important biological function in preventing amyloid formation is αB-crystallin (αBC). The molecular chaperone αBC is a small heat shock protein found in almost every tissue,^45^ and is expressed at particularly high concentrations within the eye lens where it serves to maintain lens transparency.^46^ Many studies^31,32,34,47,48^ have reported that αBC binds to a wide range of proteins to inhibit amyloid formation, including disease associated proteins such as α-synuclein,^9,32,49^ insulin,^50^ and Aβ.^31,51^ According to *in vitro* biophysical studies, the addition of αBC to solutions of α-synuclein suppresses protein aggregation both in its earliest growth stages and in later stages.^49^ The mechanism of inhibition is unclear, although the results of nuclear-magnetic resonance (NMR) spectroscopy studies suggest that αBC is able to bind α-synuclein monomers.^9,49^ Electron microscopy studies^9^ also detected the formation of amorphous α-synuclein deposits in the presence of αBC, suggesting that αBC might be able to direct pre-fibrillar α-synuclein species towards alternative pathways leading to the formation of nontoxic and biodegradable amorphous species.^9,10^

Although it is becoming increasingly evident that molecular chaperones play a crucial protective role in counteracting amyloid formation, the detailed molecular mechanisms underlying this inhibition process have not been elucidated owing to the inherent heterogeneity of aggregating protein systems in the presence of molecular chaperones, as well as the transient nature of the biomolecular interactions involved.

## 4 Dynamic oligomeric states of molecular chaperones

Under physiological conditions, many molecular chaperones, including Hsp70s and small heat shock proteins, have a propensity to self-associate to form oligomers.^52–56^ The polydispersity of molecular chaperones adds to the complexity of probing molecular chaperone–substrate interactions. Generally, the determination of the specific species interacting, the quantification of their binding affinity, and the characterisation of the population distribution and binding mechanism is difficult for interacting heterogeneous systems. It is therefore of critical importance to study the dynamics of molecular chaperone systems separately to ensure that their self-assembly behaviour can be taken into account when measuring specific interactions of these molecules with their client proteins. Elucidation of the oligomers involved in the suppression of protein aggregation is likely to yield valuable information on the molecular basis of chaperone action, the key interaction mechanisms involved, and the target amyloidogenic species. These oligomeric states of molecular chaperones have, however, proved to be particularly difficult to characterise because of the continuous interconversions between a variety of higher molecular weight species, ranging typically from dimers to dodecamers.^55,56^

**Fig. 2:**
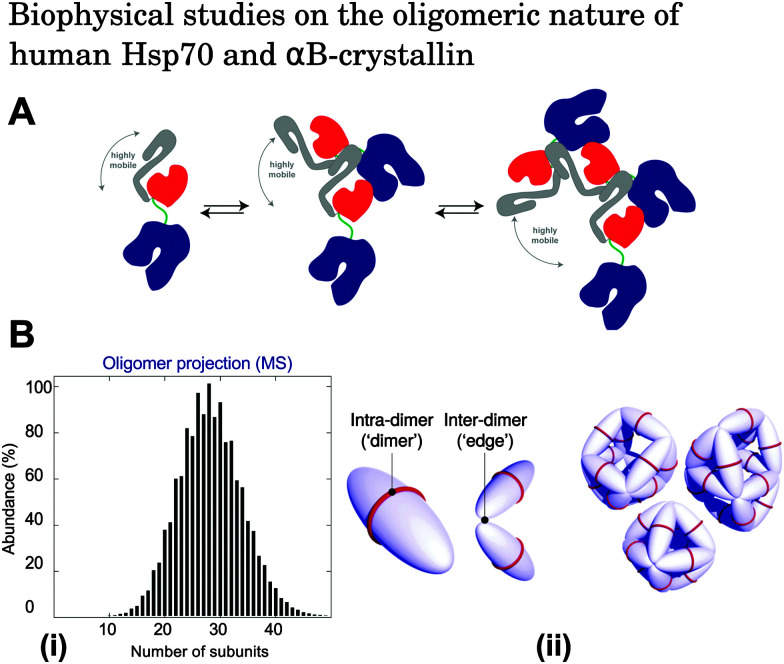
(A) Schematic illustration of the oligomerisation process of Hsp70. Components of Hsp70 are shown as follows: nucleotide binding domain (blue), substrate binding subdomain (red), helical lid subdomain (grey), and interdomain linker (green). Under physiological conditions *in vitro*, Hsp70 exists in equilibrium between monomeric, dimeric, and higher order oligomeric species.^52^ Reproduced from ref. 52. (B) (i) Oligomeric population distribution of αBC obtained by quantitative mass spectrometry measurements.^71^ Reproduced from ref. 71. (ii) Modes of interaction between αBC molecules. Intra-dimer interactions hold two αBC molecules together, and inter-dimer interactions link adjacent dimeric subunits. The result is an overall polyhedral αBC architecture. Reproduced from ref. 68.

For example, under physiological conditions *in vitro*, members of the Hsp70 family have been shown to exist as a series of monomeric, dimeric, trimeric, and higher order oligomeric species in reversible dynamic equilibrium. (Fig. 2A).^52^ Hsp70 is known to be particularly active in its monomeric form, and that its oligomerisation is regulated by the nucleotide state of chaperone–substrate binding.^52,57,58,66^ In contrast, αBC typically forms polyhedral oligomers with sizes ranging from 10-mers to 40-mers, with the most dominant species shifting between 28–32 subunits depending on the pH and solvent conditions (Fig. 2B).^53,67–70^ Although many studies have attempted to elucidate the molecular basis of αBC action,^72–75^ the effect that αBC polydispersity has on its molecular chaperone function remains unclear, with some studies suggesting that αA- and αB-crystallin exhibit maximum activity in their suboligomeric forms,^59–64^ while others have shown that intramolecularly cross-linked α-crystallin oligomers are still chaperone active.^65^ Since it is clear that the ability of molecular chaperones to bind substrates is influenced by their oligomerisation state, elucidating the dynamic behaviour of molecular chaperones is likely to be a crucial step in characterising their amyloid inhibition mechanisms and the identity of the active oligomeric species involved in their chaperone function.

## 5 Experimental challenges and biophysical approaches for the study of the mechanisms of molecular chaperone action

The polydispersity of both molecular chaperones and the variety of species generated during protein aggregation makes it challenging to identify the specific interactions occurring between these molecules. These two aspects of the problem are interconnected and are difficult to investigate separately. One way forward would be the development of rapid, high-resolution biophysical techniques capable of resolving the complete population of species within chaperone–substrate systems as well as the interactions occurring between the individual components present. Although such efforts in developing novel techniques is still in progress, several currently available biophysical approaches offer attractive possibilities for the elucidation of the oligomeric distribution of molecular chaperones as well as their interactions with amyloidogenic species. In the following sections we give a brief overview of a range of techniques used in the biophysical characterisation of molecular chaperone oligomers and their interactions with client proteins, and highlight in particular how quantitative techniques have unveiled how molecular chaperones target and bind directly to amyloid fibrils in order to counteract aggregation. A summary of the various techniques and their target species is reported in Table 1.

**Table 1:** Biophysical techniques used to characterise the oligomeric states of molecular chaperones and their interactions with amyloidogenic proteins

Target	Technique	Experimental information	Ref.
Molecular chaperone oligomers	SEC	Size distribution	52, 82 and 83
	DLS	Hydrodynamic radius	79–81
	FCS	Hydrodynamic radius	86 and 87
	IM-MS	Population distribution and bimolecular structure in the gas phase	68, 71, 88 and 89
Chaperone–protein interactions	AUC	Molecular weight distribution	31, 32, 76–78
	sm-FRET	Interactions between biomolecules in close proximity (1–10 nm)	37, 38, 84 and 85
	NMR	Solution-state: structure, interactions, and dynamics of biomolecules Solid-state: structure of insoluble biomolecules	32 and 92–95
Fibril structure and chaperone binding	SAXS & SANS	Low-resolution protein structure in solution	96
	Immuno-EM	Structure and location of proteins in cells	31, 32, 90
	Cryo-EM	Structure of biomolecules, including amyloid fibrils and oligomeric proteins	67 and 91
Chaperone–fibril interactions	QCM	Molecular binding	50 and 83

Several conventional experimental methods are available for investigating the oligomeric populations of molecular chaperones (Fig. 1F) including bulk techniques such as size-exclusion chromatography (SEC), dynamic light scattering (DLS) and fluorescence correlation spectroscopy (FCS). SEC separates single species within a heterogeneous solution according to their elution volumes, and provides information on oligomeric molecular weight distributions.^82,83^ This technique is one of the most common approaches for investigating protein size distributions and has been used to elucidate the oligomeric states of Hsp70.^52^ However, potential artefacts related to the interaction of biomolecules with the stationary phase of the column and sample dilution during the measurement should be accounted for during data analysis.

DLS and FCS allow non-invasive measurements of the diffusion coefficients of biomolecules, which in turn provide information on their hydrodynamic radii. The diffusion of analyte species is monitored as a function of time by recording light scattering or fluorescence fluctuations of the molecules of interest.^79,86,87^ These techniques have proved to be particularly effective in the sizing of homogeneous mixtures. In contrast, the evaluation of the distribution of sizes by these approaches is more difficult, since this requires the deconvolution of an average signal, which is a difficult inverse problem particularly susceptible to experimental noise.^80^ In addition, in the case of DLS, the accurate sizing of heterogeneous mixtures is complicated by the fact that the average hydrodynamic radius is dominated by larger species owing to the strong dependence of the scattering intensity on particle radius, as described by the Rayleigh formalism.^81^ As a consequence, in the context of molecular chaperones, these techniques are often used to supplement the results obtained by using other biophysical methods, such as in studies probing Hsp70 self-assembly and the interactions of Hsp70 with prion proteins.^42,52^

A more quantitative analysis of the dynamics of polydisperse oligomeric species can be achieved using mass spectrometry (MS).^53,68,71^ In particular, applications of ion mobility-mass spectrometry (IM-MS) to the study of protein complexes and amyloid oligomers in their intact, undissociated forms have provided valuable information on quaternary protein structure and topology.^88,89^ In addition, use of tandem MS and collision induced dissociation (CID) has allowed the quantification of the relative populations of oligomeric αBC as well as the energetics involved in their self-assembly.^53,68^ Typically, heterogeneous protein samples have mass spectra with overlapping peaks which arise from the different species present, making it difficult to extract quantitative information.^88^ However, this problem can be overcome using CID in which highly charged monomeric species are dissociated from aggregates prior to sample injection into the flight tube. This procedure allows the deconvolution of peaks, since the highly charged dissociated monomers give rise to a signal at low *m*/*z* whereas the complementary oligomer peaks occur at higher *m*/*z*.^88^ Although these MS techniques provide quantitative information on heterogeneous protein systems, the change of phase of the sample from solution to gas must be accounted for appropriately.

In terms of probing the interactions of molecular chaperones with their client proteins in bulk (Fig. 1D and E), analytical ultracentrifugation (AUC) is a useful method which allows separation of biomolecules according to their sedimentation coefficients, providing crucial information about their mass and size.^76,77^ A key advantage of AUC is that it is possible to characterise the binding stoichiometry and distribution of molecules in solution, including complexes of molecular chaperones and amyloidogenic proteins. For example, AUC sedimentation experiments have shown that αBC is able to bind directly to mature protein fibrils at near stoichiometric ratios.^31,32^

Single molecule techniques have recently emerged as highly effective methods for monitoring the dynamics of individual molecules in heterogeneous solutions without the need for prior separation, making it ideal for the detection of molecular chaperone interactions with prefibrillar species. Fluorescence resonance energy transfer (FRET) imaging probes the interaction of biomolecules that are spatially located at a distance shorter than 1–10 nm based on nonradiative energy transfer from a donor molecule to an acceptor molecule.^84,85^ This method therefore represents a powerful approach for detecting protein–protein interactions, and has been used to elucidate key complexes formed between both intra and extracellular molecular chaperones with aggregated species of the disease associated amyloid β(1–40) (Aβ_40_) peptide.^37,38^ The findings of these studies show that both αBC and clusterin are able to form stable complexes with Aβ_40_ oligomers spanning a range of subunit numbers, suggesting that sequestration of Aβ_40_ oligomers by these molecular chaperones plays a key role in the prevention of aberrant aggregation processes.^37,38^

Solution- and solid-state NMR spectroscopy enable the characterisation of protein conformations and allow the detection of interactions between biomolecules.^92^ For example, an NMR study on αBC and α-synuclein has shown that the addition of αBC to α-synuclein fibril solutions shifts the monomer–fibril equilibrium towards monomers.^32^ This result indicates that the binding of αBC to fibrils leads to fibril disaggregation *via* weakening of the interactions between adjacent α-synuclein molecules within mature fibrils.^32^ In addition, NMR studies are often supplemented by molecular dynamics simulations which give further insights into the structure, topology, and dynamics of proteins.^92,97–99^

Small-angle X-ray scattering (SAXS) and small-angle neutron scattering (SANS) are also highly effective and established methods for the structural characterisation of proteins in bulk. These methods do not impose a molecular weight limit as in solution NMR spectroscopy^100^ and allow solution-state measurements to be made without the need for a crystalline sample.^96^ Since the static nature of these techniques makes the detection of transient biomolecular interactions difficult,^88,89^ SAXS and SANS are commonly applied for probing the structure of fibrils and oligomers, rather than dynamic chaperone–fibril interactions.^9,101^

The techniques described above involve indirectly obtaining the size or mass of biomolecules from the measurement of their physical and chemical parameters such as sedimentation coefficient, diffusion coefficient, elution volume, and collision cross-section. The structure of complexes can also be visualised directly by imaging. In this context, electron microscopy techniques are commonly applied to the analysis of protein samples. In particular, immuno-electron microscopy and cryo-electron microscopy allow biomolecules to be imaged and provide a qualitative analysis of the different structures present, although quantification of the relative amounts remains challenging to achieve through this approach.^90^ For example, immuno-electron microscopy studies show that the mode of binding between αBC and mature α-synuclein and Aβ_42_ fibrils involves attachment of αBC along the long axis of the fibrils as well as to the fibril ends.^31,32^ This binding suggests that αBC inhibits fibril growth either by capping the reactive ends of growing fibrils and preventing elongation, or by deactivating sites which may catalyse secondary nucleation along the length of the fibril.^34,50^ Cryo-electron microscopy studies have been used to probe the quaternary structure of αBC as well as the growth of Aβ_42_ fibrils in the presence of molecular chaperones.^67,91^

**Fig. 3:**
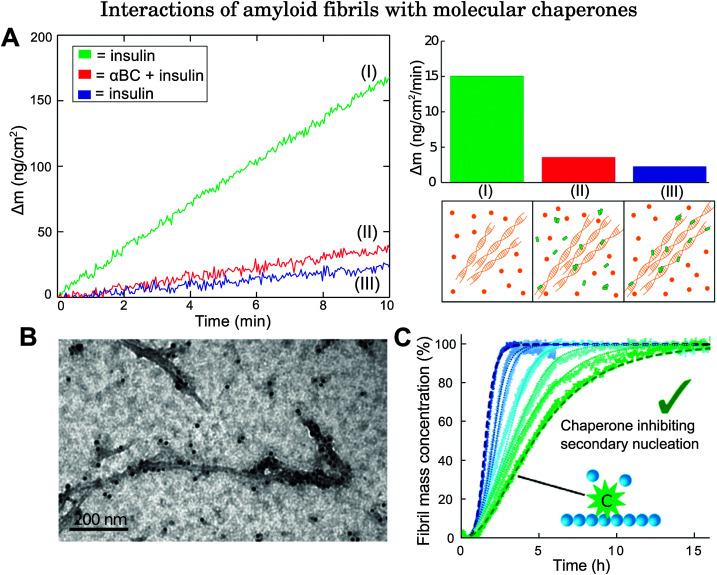
Examples of biophysical studies probing the interactions of molecular chaperones with amyloid fibrils. (A) QCM study on αBC binding to insulin fibrils. The left panel shows how the mass changes as a function of time when fibril seeds are exposed to the following conditions: green (I): fibril seeds are exposed initially to a solution of insulin; red (II): fibril seeds are then exposed to a solution of insulin and αBC, with αBC : insulin at 0.5 : 1; blue (III): fibril seeds are exposed once again to a solution of insulin only. Since fibril elongation is inhibited even after removal of the αBC solution, this implies that αBC binds directly to fibrils. Right panel: (top) Mass increase as a function of time under the three different conditions (I), (II), and (III) shown in the left panel. (Bottom) schematic illustration of (I) fibril exposure to protein monomers, (II) fibril exposure to monomers + molecular chaperones, (III) molecular chaperones bound to fibrils. Adapted from ref. 50. (B) Immuno-electron microscopy image of the molecular chaperone Brichos bound directly to Aβ_42_ fibrils. Reproduced from ref. 91. (C) ThT fluorescence study on Aβ_42_ growth in the absence of Brichos and with increasing amounts of monomer equivalents added at 10, 15, 35, 50, 75, and 100% from left (blue) to right (green). Dashed lines show the integrated rate law for Aβ_42_ growth in the absence of Brichos (blue), and the predicted reaction profile in which the rate constants for secondary nucleation processes are set to zero (green). Reproduced from ref. 91.

Quartz-crystal microbalance (QCM) and ThT fluorescence studies have illuminated additional aspects of the binding of molecular chaperones to fibrils. In QCM studies, protein fibril seeds are deposited onto the surface of a microbalance and are then exposed to a solution of protein monomers. The addition of monomeric units to the fibril seeds induces a decrease in oscillation frequency of the quartz balance. Highly sensitive measurements on molecular chaperone binding can therefore be achieved by recording the oscillation frequency as a function of time.^50,83^ By applying this technique, it has been shown that αBC is able to bind directly to insulin fibrils specifically during the fibril elongation phase, thereby inhibiting the growth of protein fibrils *via* monomer addition (Fig. 3A).^31,32,50^

ThT fluorescence studies are based on the enhanced quantum yield of ThT dye upon binding to fibrils. The time evolution of fibril formation can therefore be followed by recording the fluorescence signal of ThT. Experiments performed in the absence and presence of molecular chaperones provide evidence of the capacity of these molecules to inhibit aggregation processes. For instance, in a kinetic study conducted on Aβ_40_ growth seeded by Aβ_42_ fibrils, a decrease in fluorescence signal was observed in the presence of αBC, suggesting that the molecular chaperones interact directly with fibril seeds.^51^ Interactions between Hsp70 and α-synuclein seeds have been revealed in a similar way.^39^

In addition to the experimental techniques described above, analysis of chemical kinetics has emerged as a powerful tool for complementing experimental characterisation and for providing insights into the microscopic mechanisms of molecular chaperones acting against protein aggregation.^102^ This strategy consists of monitoring the time evolution of fibril formation (for example, by ThT fluorescence) at different protein and chaperone concentrations. The reaction profiles typically follow a sigmoidal shape which results from a highly non-linear combination of a series of microscopic events, including primary nucleation, elongation, fragmentation, and secondary nucleation. The modification of a specific microscopic event affects the reaction profiles in a characteristic manner.^102^ Therefore, kinetic analysis of the changes in the reaction profiles measured in the absence and presence of different concentrations of molecular chaperones yields information on the specific protein species targeted by molecular chaperones as well as the microscopic steps which are affected by such interactions. This method has been implemented in studies on interactions between various molecular chaperones and Aβ_42_.^91,103^ Kinetic analysis reveals that the mechanism of action which best matches the change in Aβ_42_ aggregation profile in the absence and presence of the molecular chaperone Brichos, a protein domain of approximately 100 residues, corresponds to the inhibition of secondary nucleation processes through direct binding of Brichos to Aβ_42_ fibrils (Fig. 3B and C), which thereby suppresses the generation of toxic oligomers.^91^ Similarly, it has been shown that the molecular chaperone DNAJB6 inhibits Aβ_42_ aggregation through suppressing both primary nucleation and growth events.^103^

It is clear that a variety of biophysical methods are currently available for probing molecular chaperone interactions with their client proteins through all stages during the formation of amyloid fibrils (Fig. 1D and E) as well as for investigating molecular chaperone oligomerisation separately (Fig. 1F). Most notably, quantitative biosensing techniques and applications of the theoretical aspects of protein aggregation kinetics have elucidated an important function of molecular chaperones, which is to bind directly to amyloid fibrils in order to prevent the proliferation of protein aggregates.^31,32,50,91,103^ This discovery is a crucial step forward since, although the potent inhibitory effect that molecular chaperones have on fibril generation has been well documented, the molecular mechanisms involved were previously unknown. It is becoming increasingly recognised that analysis of such systems using quantitative techniques allows the identification of the specific aggregation events that are inhibited as well as the key interactions involved, enhancing our understanding of how molecular chaperones function *in vivo*. Therefore, the development of novel quantitative biophysical methods capable of detecting individual components of a heterogeneous system of biomolecules under near physiological conditions is likely to be of critical importance in advancing our knowledge of molecular chaperone–protein interactions and the roles that they play in preventing protein aggregation.

## 6 Conclusions

The effects that molecular chaperones have on protein aggregation are increasingly well understood and evidence has emerged that they can protect against a wide variety of aberrant protein behaviour including misfolding and amyloid fibril formation processes. However, the molecular basis of chaperone action and the specific target protein species involved remain less well characterised for many systems. Elucidating the exact roles that molecular chaperones play in regulating protein aggregation is crucial for understanding the molecular events likely to be involved in the onset and progression of protein misfolding diseases. In order to address this problem and to advance our knowledge of chaperone action, it has become apparent that a detailed analysis of the oligomeric behaviour of molecular chaperones and their interactions with amyloidogenic protein species represents a key objective for biophysical studies. Progress in this direction involves the study of highly polydisperse systems in which multiple interactions occur simultaneously, a situation which arises as a consequence of the fact that molecular chaperones are commonly present as a dynamic and heterogeneous ensemble of oligomers, and are capable of interacting with a large variety of monomeric and aggregated misfolded species. Use of biophysical techniques for the characterisation of the oligomeric states of molecular chaperones, both in isolation and in the presence of their substrates, has yielded valuable insights into the molecular mechanisms underlying their function. Current and future developments in biophysical methodologies have the potential to probe increasingly complex and heterogeneous systems of aggregates of client proteins and molecular chaperone oligomers, and to open up the possibilities for their quantitative characterisation.

## Supplementary Material
